# Clinical Tests and Functional Capacity in Office Workers With Nonspecific Chronic Low Back Pain Between Mild and Moderate Pain Intensity: A Comparative Study

**DOI:** 10.1155/prm/3908009

**Published:** 2026-04-26

**Authors:** Supartina Hakim, Prasert Sakulsriprasert, Roongtiwa Vachalathiti

**Affiliations:** ^1^ Faculty of Physical Therapy, Mahidol University, Nakhon Pathom, 73170, Thailand, mahidol.ac.th

**Keywords:** clinical tests, functional capacity, low back pain, severity, straight leg raising

## Abstract

**Objectives:**

This study aimed to compare the clinical tests and functional capacity based on mild and moderate pain intensity in the office workers with nonspecific chronic low back pain (NSCLBP).

**Methods:**

Eighty participants, divided into mild and moderate pain groups based on pain intensity (40 each group), were recruited into this cross‐sectional study. Passive lumbar extension test (PLET) and straight leg raising (SLR) were assessed as the clinical tests. The five‐time sit‐to‐stand (5STS) and two‐minute step test (TMST) were measured as the functional capacity tests. The Mann–Whitney U test and chi‐square test were used for between‐group comparisons.

**Results:**

The findings of this study demonstrated that the office workers with NSCLBP in both mild and moderate pain groups had similar results in degrees of SLR and positive results of PLET, as well as in 5STS and TMST (*p* value > 0.05).

**Conclusions:**

This study suggested that the severity of NSCLBP described as mild or moderate pain intensity could not differentiate PLET, SLR, 5STS, and TMST. Individuals with NSCLBP also have positive results in clinical and functional tests. Therefore, PLET, SLR, and functional capacity should be tested in both mild and moderate NSCLBP.

## 1. Introduction

Low back pain (LBP) is one of the common musculoskeletal disorders and the leading cause of disability in the world [1]. Most of the LBP population develop chronic LBP defining as a duration lasting for 3 months or longer [1, 2]. Approximately 75%–85% of LBP cases are classified as nonspecific chronic low back pain (NSCLBP), which occurs from overuse and dysfunction of surrounding spinal tissues [3].

Nowadays, one of the common causes of NSCLBP in office workers is prolonged sitting and poor sitting posture [4]. The sitting time of office workers on weekdays was reported to be 5–8 h per day in several countries [5–7]. Previous studies have found the association between prolonged sitting of more than 8 h per day and the prevalence of NSCLBP ranging from 20% to 44% in a year in the working population [4, 8]. According to the 2005 Indonesian employee health profile, 16% of employees suffered from this condition [6]. A previous study reported that the one‐year prevalence of musculoskeletal symptoms among Indonesian office workers is about 60% [9]. Office workers with NSCLBP in industrialized countries are associated with high expenses due to the increased compensation costs and the absence from work or loss of productivity [6]. It causes activity limitations in office workers, which is an important reason for seeking physician consultation.

LBP could be classified by several methods. Symptom‐based classification is often used, which describes the classification according to pain severity: mild (Level 1–3), moderate (Level 4–6), and severe (Level 7–10) [10, 11]. A numeric rating scale (NRS) is a method that is frequently used clinically for pain rating, which ranges from 0 to 10, which 0 representing “No Pain” and 10 “Unbearable Pain.” The transition from mild to moderate pain may seem incremental, but it often represents critical psychological and behavioral aspects. The primary reason this distinction is expected to differentiate functional capacity lies in the fear‐avoidance, the deconditioning loop, and neurological sensitization [10, 12, 13].

In office workers, the static sitting posture leads to compression at the anterior part of the disk and an opening in the facet joints during the flexion posture in the sagittal plane [14]. Any excessive forward spinal flexion that may stress the posterior ligaments, facet joint and capsule, intervertebral disk, and paraspinal muscles would increase pain [15]. These conditions can lead to lumbar segmental instability (LSI), that is, the estimated prevalence of LSI reported 12% of patients attending physical therapy for NSCLBP [16], while 57% suspected of having LSI [17]. Several clinical tests were routinely employed to diagnose LSI. The passive lumbar extension test (PLET) is a valid and reliable test to indicate LSI in patients with LBP [18]. Kasai et al. in 2006 found that the PLET was the most accurate clinical test for detecting clinical lumbar instability, which showed validity with high sensitivity (0.84, 95% CI: 0.7–0.93), specificity (0.90, 95% CI: 0.82–0.95), and positive likelihood ratio (8.8, 95% CI: 4.5–17.3) [19].

The straight leg raising (SLR) test is one of the most commonly performed tests in clinical practice for neural tissue used to test movement and mechanical sensitivity of the lumbosacral neural structures and their distal extension [20]. The SLR test is widely used in the assessment of altered nerve mechanosensitivity [21]. The SLR test has been used in all cases of back and leg pain that include nerve root compression and tension [20]. Increased tension in the musculoskeletal and nervous system would be one contributing factor to musculoskeletal dysfunction and pain [22]. Therefore, assessments of their surrounding structures in low back and the lumbosacral neural structures would be beneficial.

Among office workers, transitioning between sitting and standing is an essential common daily activity for functional independence with the movement performed, on average, 60 times a day [23]. Sit‐to‐stand (STS) provides information on functional independence in daily activities in office workers with NSCLBP who might perform STS more slowly than a healthy person. STS showed adequate responsiveness to detect change over time in people with chronic LBP [24]. A higher level of pain intensity would have a negative influence in sit‐to‐stand performance, which is one functional capacity task [25]. In the study by Shum et al., patients with LBP had significantly reduced trunk velocity and longer duration of STS [26]. In addition, functional capacity is also used to express the ability to perform activities of daily living [27]. The two‐minute step test (TMST) is one of the assessments of aerobic function and dynamic balance in older people and is widely used in clinical practice. The TMST was first designed for using in the older population; however, it is now recognized for broader ability to predict functional capacity in all individuals. The STS and TMST involve trunk and lower limb muscle functions, which indicate the strength and endurance of trunk and lower limb muscles. The assessment of the functional capacity classified by pain intensity would be valuable information in clinical practice reflecting the performance of patients with NSCLBP.

Without severe pain intensity, the office workers would be able to continue their daily work. Therefore, the purpose of this study was to compare clinical tests and functional capacity based on mild and moderate pain intensity in office workers with NSCLBP. We hypothesized that the office workers with mild pain would have better clinical tests and functional capacities compared to those with moderate pain.

## 2. Methods

### 2.1. Study Design, Setting, and Subjects

**FIGURE 1:**
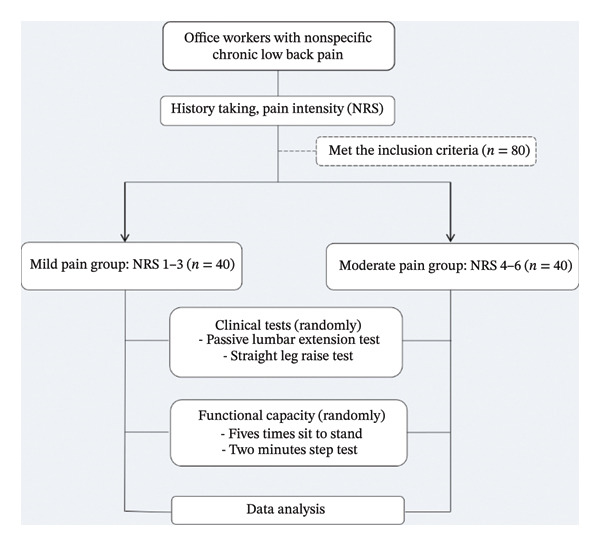
Flowchart of the study, note: NRS = numeric rating scale.

This study was a cross‐sectional study design comparing clinical tests and functional capacity based on the level of pain intensity between mild and moderate pain among office workers with NSCLBP, as shown in Figure 1. NRS from 0 to 10 representing pain intensity verbally was used in this study. The office workers with NSCLBP reported their pain intensity on the worst movement or activity on the tested day by verbal expression for NRS. The level of mild pain intensity was 1–3 out of 10, and the level of moderate pain was 4–6 out of 10 according to NRS scoring in the last 24 h. We selected two companies in Makassar, Indonesia, with a similar working style to office workers. The inclusion criteria were LBP at least three months without radicular pain, working at least 5 h per day, and working experience of at least 2 years. The pain level of each participant was assessed by the assistant, who was blinded from the tests. Exclusion criteria were neurological or cardiovascular diseases that affect activities in daily life, symptoms from spinal infection or spinal tumors, LBP with radiating pain, history of spine or lower extremity surgery, problems in lower extremity, and pregnancy or menstruation. The required sample size was calculated from our pilot data of 10 subjects using the G^∗^Power 3.1.9.4 program with alpha level 0.05, power 0.80, and effect size (ES) value 0.64 from the SLR test, which were determined based on mean differences and standard deviations. The calculated sample size was 80 participants totally or 40 participants for either the mild or moderate pain group.

All participants received detailed information about the study and signed an informed consent form before participation. The data were collected from October to December 2019. This study was conducted in accordance with the Declaration of Helsinki and was approved by the Ethics Committee of the Mahidol University Central Institutional Review Board (MU‐CIRB), No. 2019/147.1809.

### 2.2. Clinical Tests

#### 2.2.1. PLET

The participants were in the prone position, and the researcher elevated both legs to a height of about 30 cm from a couch while maintaining the knees straight and gently pulling the legs. Before testing, the researcher marked the level of height 30 cm on the wall by using a mark tape and for both legs’ elevation to the indicated level. The test was considered positive when the participant complained of pain or felt heavy on the low back during the elevation of both legs and pain disappeared when their legs returned to the initial position [18, 19]. To ensure procedural consistency and data validity, the assessor completed a standardized PLET training session prior to data collection.

#### 2.2.2. SLR Test

Each leg was tested individually with the dominant leg being tested first. The leg was tested by three tests: kicking the ball, drawing number eight on the floor, and picking a rubber on the floor. The dominant leg was the leg that performed at least two tests.

In the SLR test, a participant was positioned in supine without a pillow under his/her head, and the hip was on the neutral position and the knee extension. SLR was measured with a bubble inclinometer. The bubble inclinometer was set to zero point on the table prior to being placed on the tibial crest below the tibial tubercle. The researcher lifted the participant′s leg with knee straight by flexing at the hip until the subject was feeling of discomfort/unbearable in the back or posterior aspect of the leg, and the researcher reached end‐range resistance. The measured value was then recorded in degrees [20, 21, 28]. The intratester reliability (ICC_3,1_) of SLR measurement was 0.81.

### 2.3. Functional Capacity Tests

Each participant performed two functional capacity tests: 5STS and TMST. During the tests, all participants wore comfortable clothes and canvas shoes. They were explained the test protocols and observed demonstration, with a question‐and‐answer session provided if needed [29]. To ensure procedural consistency and data validity, the assessor completed a standardized training session prior to data collection.

#### 2.3.1. 5STS Test

The starting position of 5STS is the participants sit in the middle of the chair, back straight without support, feet flat on the floor, and both arms folded together across the chest. They were then asked to rise to full stand and return to the sitting position as fast as possible five times continuously [29]. They performed three trials, and the shortest time spent to complete the 5STS was used.

#### 2.3.2. TMST

The researcher measured the stepping height of each participant, which was equal to the mid‐thigh level, halfway between the iliac crest and patella, and marked that level on the wall. The starting position was standing, and the participant was then instructed to step by alternately moving the knee up to the marked level, beginning with the right knee and continuing to step for 2 min. The researcher counted the number of times the right knee successfully reached the marked level. The participant was allowed to place one hand on a table for balance assistance if needed [29]. They performed this test only once. However, before data collection, the researcher allowed each participant a short practice.

A rest interval of 2 min was provided to reduce fatigue between each test. The researcher asked all participants every time before starting the next tests to reassure that all participants had no carry‐over symptoms. After two tests, each participant was asked to slowly walk for a few minutes to cool down.

All tests were performed on the same day. All participants were assessed for the clinical tests first and then measured for functional capacity tests in the last. The PLET and SLR were randomly assessed by using a sealed envelope. The assessor was blinded to whether participants were in the mild or moderate pain group. The 5STS and TMST were also randomly performed. The total duration of all tests was approximately 30–45 min.

### 2.4. Statistical Analysis

Data were analyzed by using SPSS Version 23. The statistical significance level was set at a *p* value of < 0.05. The Kolmogorov–Smirnov goodness‐of‐fit test was used to test for the distribution of the data. Descriptive statistics were used to present demographic data, such as age, sex, body mass index (BMI), and working characteristics of office workers. The mean and standard deviation were calculated for all continuous data and frequencies for category variables. If data were of normal distribution, the independent *t* test was used to compare the difference between mild and moderate pain intensity groups. If data were not normally distributed, the Mann–Whitney *U* test was performed. For categorical data, positive and negative results, the chi‐square test was used to determine the difference between mild and moderate pain groups. For determining the effects of sex, age, weight, and height as confounders (independent variables) on clinical tests and functional capacity tests (dependent variables), the linear regression analysis, enter method, was used.

## 3. Results

Eighty participants were recruited in the study and divided based on pain intensity, forty participants in each group: 21 males and 19 females in the mild pain group and 12 males and 28 females in the moderate pain group. The demographic data of both groups were similar in age, weight, BMI, duration of symptoms, and working experience, as shown in Table 1. The details of age range, pain intensity, time onset, BMI, routine sport, or exercise are reported in Table 2. No significant differences in any characteristic were found between the two groups, except for height and sex.

**TABLE 1:** Characteristics of participants (*N* = 80).

Variables	Group (mean ± SD)		Range		*p* value
	Mild (*n* = 40)	Moderate (*n* = 40)	Mild	Moderate	
Age (years)	33.75 ± 7.21	32.48 ± 6.76	25–45	25–45	0.432
Height (cm)	162.52 ± 9.34	158.55 ± 6.02	145–182	148–171	0.036
Weight (kg)	64.28 ± 11.74	62.80 ± 10.56	40–84	43–84	0.556
BMI (kg/m^2^)	24.30 ± 3.78	24.92 ± 3.62	15.7–32.5	19.0–32.0	0.460
NSCLBP duration (months)	16.65 ± 16.92	16.80 ± 14.83	1–60	1–60	0.580
Working experiences (years)	8.75 ± 7.59	7.50 ± 6.49	2–28	2–25	0.782

**TABLE 2:** Details of each characteristic.

**Variables**	**Number of participants**		**Total**
	**Mild (*n* = 40)**	**Moderate (*n* = 40)**	

Age (years)			
25–35	24 (60%)	28 (70%)	52 (65%)
36–45	16 (40%)	12 (30%)	28 (35%)
Pain intensity	2.13 ± 0.88	5.15 ± 0.83	80 (100%)
Time onset (pain)			
3–6 months	14 (35%)	10 (25%)	24 (30%)
7–12 months	13 (32.5%)	17 (42.5%)	30 (37.5%)
1–2 years	6 (15%)	7 (17.5%)	13 (16.3%)
2–3 years	3 (7.5%)	3 (7.5%)	6 (7.5%)
3–4 years	1 (2.5%)	1 (2.5%)	2 (2.5%)
4–5 years	3 (7.5%)	2 (5%)	5 (6.3%)
BMI (kg/m^2^) [27]			
Underweight (< 18.5)	3 (7.5%)	—	3 (7.5%)
Normal (18.5–24.90)	19 (47.5%)	20 (50%)	39 (48.8%)
Overweight (25–29.90)	15 (37.5%)	17 (42.5%)	32 (40%)
Obesity (> 30)	3 (7.5%)	3 (7.5%)	6 (7.5%)
Sport/exercise			
Routine sport/exercise	30 (75%)	31 (77.5%)	61 (76.3%)
No sport/exercise	10 (25%)	9 (22.5%)	19 (23.8%)
Dominant foot (right/left)	37 (92.5%)/3 (7.5%)	38 (95%)/2 (5%)	75 (93.75%)/5 (6.25%)

Table 3 demonstrates the findings of clinical tests and functional capacity for mild and moderate pain groups. The PLET was found positive for 10 out of 40 participants in the mild pain group and positive for 14 out of 40 in the moderate pain group. The chi‐square test indicated no significant difference between mild and moderate pain intensity groups (*X* ^2^ = 0.9524, *p* = 0.33, ES = 0.23).

**TABLE 3:** Clinical tests and functional capacity tests.

Variables	Group (mean ± SD)		Range		*p* value	ES
	Mild	Moderate	Mild	Moderate		
PLET	Negative 30 Positive 10	Negative 26 Positive 14	—	—	0.33^∗^	0.23
SLR dominant (degrees)	78.43 ± 12.29	83.38 ± 14.30	40–100	65–120	0.314^#^	0.37
SLR nondominant (degrees)	82.62 ± 12.76	83.13 ± 10.36	45–110	65–110	0.961^#^	0.04
5STS (seconds)	12.41 ± 2.40	11.56 ± 1.754	8–18	7–15	0.201^#^	0.40
TMST (steps)	78.42 ± 13.39	77.20 ± 17.69	32–105	25–105	0.728	0.08

*Note:* 5STS = five times sit‐to‐stand.

Abbreviations: ES = effect size, PLET = passive lumbar extension test, SLR = straight leg raising, TMST = two‐minute step test.

^#^ *p* value from Mann–Whitney U test.

^∗^ *p* value from chi‐square test.

The SLR test results of dominant legs ranged from 40° to 100° in the mild pain group and 65° to 120° in the moderate pain group, while the SLR test results of nondominant legs ranged from 45° to 110° in the mild pain group and 65° to 110° in the moderate pain group. There were no significant differences between groups in either the dominant or nondominant leg (*p* = 0.314, ES = 0.37 and *p* = 0.961, ES = 0.04, respectively). Table 4 demonstrates the number of participants in subgroups of SLR 30°–70° and greater than 70°.

**TABLE 4:** Subgroups of SLR test.

No	Variables	Number of participants		Total
		Mild (*N* = 40)	Moderate (*N* = 40)	
Straight leg raising dominant				
1	30–70 (degrees)	10 (25%)	11 (27.5%)	21 (26.3%)
2	> 70 (degrees)	30 (75%)	29 (72.5%)	59 (73.8%)
Straight leg raising nondominant				
1	30–70 (degrees)	7 (17.5%)	6 (15%)	13 (16.3%)
2	> 70 (degrees)	33 (82.5%)	34 (85%)	67 (83.8%)

Means and standard deviations of time used for 5STS and number of steps in TMST for both groups are also presented in Table 3. No significant difference in any functional capacity test was found between mild and moderate pain groups (*p* = 0.201, ES = 0.40 and *p* = 0.728, ES = 0.08, respectively).

Regression analysis indicated that demographic variables, including sex, age, weight, and height, were not significant confounders for any clinical or functional capacity tests (all *p* value > 0.05).

## 4. Discussion

The objectives of this study were to compare clinical tests and functional capacity between office workers with mild and moderate NSCLBP. No significant difference was indicated between groups either on clinical tests or functional capacity.

Each participant sat for at least 5 h a day during work. This indicated that even though adult office workers were in a period of working life, they could also approach risk of LBP. The study of Butte et al. in 2023 demonstrated a relationship between high amounts of sitting and postural compensations that may lead to LBP [30]. Sitting for a long time acted as a constant load, leading to gradual maladaptation to back muscles, the lumbar spine, and other associated passive structures. Therefore, the prevention of NSCLBP and health promotion must be strongly recommended to decrease chronicity of the LBP condition, which include young and middle‐aged adults.

For PLET, a prevalence of 25% (10 out of 40 participants) indicated LSI in the mild pain group, while 35% (14 out of 40 participants) demonstrated LSI in the moderate pain group. However, these positive numbers could not significantly differentiate between mild and moderate pain intensity in individuals with NSCLBP. The positive result of PLET might be owing to the gradual adaptation of passive structures such as lumbar spine and its associated structures after sustained position, which happens continuously over the period of time caused by prolonged sitting, which can be explained by the creep phenomenon, which is defined as the tendency of a solid material to slowly deform permanently under the influence of mechanical stresses [31, 32]. Regarding the creep phenomenon, it might explain the findings of the positive results of PLET that were evident in both groups of mild and moderate pain intensity.

Abbott et al. in 2005 [16] reported that the prevalence of lumbar instability in NSCLBP was 12% by using the objective examination test as passive accessory intervertebral movement and passive physiological intervertebral movement tests. Puntumetakul et al. in 2015 [33] reported the prevalence of 13% lumbar instability among Thai rice farmers. Rabin in 2013 showed that the prevalence instability in NSCLBP ranged from 38% to 41% when measured using PLET [34]. From the findings of PLET in the present study, the researchers, therefore, recommended to use PLET as one of the clinical tests for checking LSI in NSCLBP in both mild and moderate pain intensity groups, which might be found positive.

The average value of SLR was 80° for both groups, which indicated the tightness of hamstring muscle [28]. However, when we did subgroup analysis of SLR into two ranges: 30–70° and greater than 70° (Table 4), it was found that 15% to 27.5% of the office workers with NSCLBP had tension even though they did not have any complaint on their legs. Therefore, the SLR test is a useful clinical test to detect neural mechanosensitivity in individuals with NSCLBP [35]. The indifferent result of SLR between mild and moderate pain groups might be due to the fact that SLR was not affected by pain intensity perceived individually. In addition, the primary source of pain in this study was not from the neural structure, but musculoskeletal instead. Therefore, the measurement of SLR was not sensitive enough to classify mild versus moderate LBP in individuals with nonspecific LBP. The gradual adaptation of active and passive structures among all individuals in this study caused by prolonged sitting might change the neural mechanosensitivity [31].

For functional capacity, the results showed no significant difference in either 5STS or TMST between the two groups. These findings did not support our hypotheses. According to the result in the present study, the level of mild and moderate pain intensity did not directly affect the ability to perform 5STS and TMST in office workers with NSCLBP. The indifferent results between the two groups might reflect that pain intensity is the subjective domain toward self‐perception, but it lacks the association with functional capacity since the deterioration of function takes a period of time for progression. A nonsignificant difference in functional tests may be because the participants were able to work in the office even though they had moderate pain.

Interestingly, the times spent for 5STS in both mild (12.41 s) and moderate (11.56 s) pain groups in the present study were longer than times spent by healthy adults aged between 30 and 58 years (8.08 s) in the study of Vachalathiti et al. [36]. Moreover, 5STS values in NSCLBP in this study were almost the same as the values in healthy individuals with age range between 60 and 69 years (11.4 s) and between 70 and 79 years (12.6 s) in Bohannon’s study [37].

For TMST, the averaged values of mild (78.42 steps) and moderate (77.20 steps) pain groups in this study were less than those of older people aged 70–80 years, showing an averaged value of 84.73 steps for older men and 81.68 steps for older women [38]. This result might give us the awareness of worsening of dynamic control in individuals with NSCLBP [39–41].

The decreased level of functional capacity in individuals with NSCLBP found in this study might be due to poor trunk motor control as shown by longer time spent in the 5STS and fewer steps counted in the TMST. These tests require both back and lower extremity to perform in the combination which need their strength and motor control to accomplish [39–42]. The rest interval provided between tests and the reassurance questions asked to every subject have been done as a standardized method. The findings of the present study could be implied that the office workers with NSCLBP had declined their functional capacity in 5STS and TMST. Therefore, clinicians should assess the functional capacity in individuals with NSCLBP for better understanding of the physical condition and promoting good healthcare for NSCLBP.

This study may have some limitations. First, the sample size was too low to detect the difference between the mild and moderate groups, even though we had already calculated the sample size from the pilot study. According to the ESs of each parameter, around 148–16,306 subjects are needed. In addition, NSCLBP comprises physical and biopsychosocial factors. This study did not investigate the biopsychosocial dimension. The results of this study could not be generalized to all patients with NSCLBP, in particular for NSCLBP with severe pain intensity. In addition, some dimensions need to be considered regarding the quality of pain, frequency, and functional limitation. The aforementioned further information warrants formal investigation in future studies. Finally, this study utilized only a single TMST trial, and the findings consistently reflect reduced aerobic and functional capacity, aligning with the observed clinical presentations of NSCLBP [43].

### 4.1. Study Strength and Future Research

The current literature provides limited insight into how pain intensity levels in NSCLBP subgroups relate to clinical and functional capacity. This study investigated office workers with mild‐to‐moderate NSCLBP, who remained active in the workforce, offering a pragmatic representation of real‐world occupational conditions. Our findings highlight the necessity for targeted activity and exercise interventions to promote functional capacity and reduce clinical risks. Future research should prioritize functional capacity and behavioral awareness training to mitigate the socioeconomic and personal burden of NSCLBP among office workers.

To enhance generalizability, future studies should incorporate cohorts with severe NSCLBP, radicular symptoms, and specific spinal pathologies. Furthermore, longitudinal physiological and biomechanical investigations are warranted to elucidate the underlying mechanisms of NSCLBP pathophysiology. Finally, establishing diagnostic cut‐off points for the clinical and functional variables examined in this study is essential to effectively stratify LBP severity and guide targeted clinical decision‐making.

## 5. Conclusion

The findings of this study provided evidence that clinical tests and functional capacity were not directly influenced by pain intensity in office workers with NSCLBP. This study suggested that the severity of LBP described as mild or moderate pain intensity could not differentiate the values of SLR, numbers of positive PLET, or functional capacity. Moreover, PLET can be recommended for checking instability in individuals with NSCLBP by which positive results can be found in mild and moderate pain groups. Functional capacity should be assessed and promoted in NSCLBP to prevent health deterioration.

## Author Contributions

All authors participated in the conception and design. Supartina Hakim collected the data and performed statistical analysis. Prasert Sakulsriprasert and Roongtiwa Vachalathiti interpreted the data. Supartina Hakim wrote the first draft of the manuscript. Roongtiwa Vachalathiti critically revised the manuscript.

## Funding

The authors received no specific funding for this work.

## Disclosure

All authors read and approved the final manuscript.

## Ethics Statement

This study was approved by the Ethics Committee of the Mahidol University Central Institutional Review Board (MU‐CIRB), No. 2019/147.1809.

## Conflicts of Interest

The authors declare no conflicts of interest.

## Data Availability

The data that support the findings of this study are available from the corresponding author upon reasonable request.
